# Current Status of Catheter-based Mitral Valve Replacement

**DOI:** 10.1007/s11886-021-01524-0

**Published:** 2021-07-01

**Authors:** Elias Rawish, Tobias Schmidt, Ingo Eitel, Christian Frerker

**Affiliations:** 1 University Hospital Schleswig-Holstein, Medical Clinic II, University Heart Center Lübeck, Lübeck, Germany; 2 DZHK (German Centre for Cardiovascular Research), partner site Hamburg/Kiel/Lübeck, Lübeck, Germany

**Keywords:** Transcatheter mitral valve replacement, TMVR, Mitral regurgitation, Transcatheter cardiac therapeutics, Transcatheter mitral intervention, Transcatheter mitral implantation

## Abstract

**Purpose of review:**

Transcatheter mitral valve replacement (TMVR) has been developed to address the need for an alternative therapeutic option to surgery in patients suffering from severe mitral regurgitation who are at high surgical risk. The present review illustrated the state-of-the-art of catheter-based mitral valve replacement evaluating technical characteristics and early clinical experience of different devices to outline prospects and challenges of TMVR.

**Recent findings:**

Several devices are currently under clinical assessment. Early experience has demonstrated high procedural success of TMVR. However, TMVR faces several possible hurdles such as left ventricular outflow tract obstruction (LVOTO) after prosthesis deployment, access site complications, and thrombotic risk requiring anticoagulatory therapy.

**Summary:**

Future studies should assess long-term prosthesis stability, optimal anticoagulation regime, and occurrence of paravalvular leakage. The development of smaller TMVR prostheses suitable for transseptal implantation could overcome bleeding complications. In perspective, TMVR may emerge to a clinically relevant therapeutic approach for patients with severe MR at high surgical risk.

## Introduction

Mitral regurgitation (MR) is the most common valvular disease in the adult population affecting almost 10% of individuals over the age of 75 years [1]. Although conventional mitral valve surgery represents the first-line therapy for patients with symptomatic severe MR, up to 50% of those affected are not referred for surgery due to high risks from age and comorbidities [2]. An overall 5-year mortality rate of 50% was reported in unoperated patients [3], emphasizing the pressing requirement for minimally invasive procedures in treatment of severe MR. Indeed, different transcatheter mitral valve devices have emerged for the treatment of MR in high-risk or inoperable patients. The edge-to-edge leaflet repair system MitraClip (Abbott Laboratories, Abbott Park, IL, USA) constitutes the most frequent applied transcatheter mitral valve repair (TMVr) technique for patients with degenerative mitral regurgitation and functional mitral regurgitation. Even though the COAPT trial yielded a lower mortality and lower hospitalization rate due to heart failure in patients treated with TMVr than best medical therapy alone [4], the MITRA-FR trial showed opposing results [5], which have been attributed to different inclusion criteria, as COAPT enrolled a subset of patients who had more severe MR and less advanced left ventricular (LV) failure [6]. Beyond the decisive importance of patient selection, TMVr systems, however, have further significant limitations, including challenging application due to the presence of advanced valve leaflet damage or excessive ventricular remodeling and subsequent persistence or reoccurrence of MR [7]. Transcatheter mitral valve replacement (TMVR) systems may overcome these limitations, as they may offer a “one valve fits all” concept with more predictable reduction of MR, and less technically demanding procedures [8]. Thus, the present review will address the current state-of-the-art of catheter-based mitral valve replacement evaluating technical characteristics and early clinical experience of different TMVR devices in patients with native MV to outline prospects and challenges of TMVR.

## Anatomical structure and subsequent prosthesis design challenges

### Prothesis anchoring and sealing

The anatomy of the mitral valve (MV) is considerably more complex than that of the aortic valve (AV). The AV consists of three leaflets in the tube-shaped ascending aorta, allowing straightforward percutaneous access, thereby severe calcification due to calcified aortic valve disease enables the exclusive use of radial forces to seat transcatheter aortic valve replacement (TAVR) devices. By contrast, the MV apparatus is a dynamic three-dimensional (3D) system, consisting of the D- or saddle-shaped rigid annulus, the anterior MV leaflet which is rounded and occupies one-third of the MV annulus, and the semilunar shaped posterior MV leaflet that occupies two-thirds of the MV annulus circumference [9]. These leaflets are attached to the collagenous chordae tendineae that bulge from the papillary muscles, constituting the sub-valvular apparatus. Valve fixation in TMVR cannot solely depend on radial forces similar to in TAVR due to a frequent lack of calcification and a dynamic annular region, thus different anchoring techniques have been proposed [10, 11]. These include the usage of counteracting axial forces by tethers (Tendyne valve, Abbott Laboratories, Abbott Park, IL, USA), grasping of native leaflets by ventricular anchors (Tiara valve; Neovasc, Richmond, BC, Canada), atrial and ventricular flanges that latch the MV annulus and leaflets (CardiAQ, Edwards Lifesciences, Irvine, CA, USA), champagne cork-like effect enabling adequate radial forces for fixation (Intrepid valve; Medtronic, Minneapolis, MN, USA), and formation of a landing zone by implanting an anchor ring (HighLife, HighLife Medical, Paris, France) [11]. The delineated anchoring techniques are intended not only to protect against prothesis dislocation but also to prevent paravalvular leakage following valve implantation.

### Delivery approach

Although a percutaneous transfemoral venous approach with subsequent transseptal access is considered less invasive, and thus is the preferred approach for TMVR, its technical implementation is highly challenging because the delivery system has to handle a nearly right-angle bend within a fairly small space to reach the MV following transseptal puncturing. Therefore, especially early TMVR systems have been developed to deliver the valve prosthesis transapically. However, higher complication rates and poorer outcomes have been associated with the transapical approach in TAVR compared with transfemoral access [12, 13]. While transapical patients had a higher base morbidity than transfemoral patients, propensity-matching have revealed an association of transapical approach with increased mortality due to increased bleeding complications and reduced recovery of LV function [13]. Hence, developers are currently focusing on the design of transeptally applicable TMVR prostheses suitable for smaller lumen catheters.

### Left ventricular outflow tract obstruction

Left ventricular outflow tract obstruction (LVOTO) has been reported following surgical implantation of valve prothesis and annuloplasty rings [14] but also after surgical mitral valve repair [15]. LVOTO represents a life-threatening complication with 62% in-hospital mortality [16], and has been described after transcatheter mitral valve-in-ring procedures as well [17]. Thus, considering the larger prothesis size in TMVR, LVOTO constitutes a decisive design obstacle [18]. TMVR-induced LVOTO has been related to two mechanisms: Fixed obstruction, which follows anterior MV leaflet shifting toward the interventricular septum by the TMVR prosthesis, creating a narrowed and elongated neo-LVOT [19]; and dynamic obstruction, which is predisposed by long anterior MV leaflet and relies on Bernoulli forces rendered by the narrowed neo-LVOT that pull the anterior MV leaflet toward the interventricular septum during systole [20, 21]. The Neo-LVOT area should be predicted by pre-procedural time-resolved computed tomography (CT) to identify patients at increased anatomical risk for LVOTO. For that approach, end-systolic measurements are commonly used [22]. However, Meduri et al. have recently shown that multiphase and particularly early systolic neo-LVOT assessment is superior to end-systolic estimation in determining risk of LVOTO, significantly increasing patient eligibility, as over one-half of patients previously screen-failed were eligible for treatment using early systolic assessment [23]. Thus, further prospective studies should validate this method because it could allow a much broader treatment population. With respect to preventive strategies against LVOTO in high-risk patients, prophylactic alcohol septal ablation has been reported to be effective in some cases [24]. However, it is not anatomically feasible in all patients and can be accompanied by further reduction of LV function, need for permanent pacemaker, and ventricular septal defects [25]. In addition, septal ablation delays TMVR by 4 to 6 weeks. Thus, radiofrequency ablation-based septal correction to prevent iatrogenic LVOTO (SCORPION) procedure has recently been evaluated to increase LVOT diameter prior to TMVR: While three out of three patients underwent successful SCORPION, all patients developed high-grade atrioventricular block requiring pacemaker implantation [26]. However, ventricular septal defect did not occur [26].

Another promising approach is the intentional laceration of the anterior MV leaflet to prevent the LVOTO (LAMPOON) procedure, which aims to preserve the anterior MV leaflet from covering the open cells of the TMVR prosthesis [21]. Indeed, a recent clinical trial yielded the feasibility of LAMPOON to prevent LVOTO from TMVR in patients who were otherwise not treatable, with acceptable safety [21]. However, this technically challenging approach is only suitable to TMVR devices with an open-cell design, that is, SAPIEN 3 valve (Edwards Lifesciences, Irvine, CA, USA). Owing to the closed-cell design, laceration of the anterior MV leaflet would not reduce the risk of LVOTO in most novel TMVR devices [23]. While the LAMPOON procedure constitutes a transseptal approach, the balloon assisted translocation of the mitral anterior leaflet to prevent left ventricular outflow obstruction (BATMAN) technique has similar principles to the LAMPOON procedure but only can be performed by using transapical access. Penetrating and ballooning the anterior mitral leaflet results in formation of a hole and posterior translocation of the anterior mitral leaflet to deploy the TMVR valve [27]. However, to date, the BATMAN procedure can only be applied under cardiopulmonary bypass.

## Pre- and periprocedural aspects

### Multimodality imaging

Transthoracic echocardiography (TTE) constitutes the primary imaging modality for the diagnosis and assessment of the mechanism and severity of MR [11]. However, electrocardiogram (ECG)-gated cardiac CT represents the preferred imaging modality for preprocedural TMVR evaluation to confirm patient eligibility based on anatomic characteristics, and predict fluoroscopic angulation as well as access location [28]. The evaluation includes the morphology of the mitral annulus, extent of calcifications in the mitral ring, the subvalvular apparatus, size and shape of left atrium and ventricle, and mitro-aortic angle, thereby simulation of the procedure may help to predict possible compilations [7]. Intra-procedural fluoroscopy guides vascular access and catheter manipulation in the cardiac chambers, while (3D) transesophageal echocardiography (TEE) is the primary modality for device deployment, which have been extensively reviewed elsewhere [10]. Recently, echocardiographic–CT scan fusion has emerged as an innovative tool, enabling the visualization of both image modalities in the same visual perspective on the echo screen during TMVR procedure through fusion of pre-interventional CT scan and periprocedural 3D-TEE [29]. Coisne et al. concluded that this technique may be beneficial in catheter crossing of the annular plane avoiding the subvalvular apparatus, position assessment of the delivery system during final deployment, and LVOT impact after deployment of recapturable and retrievable prostheses [29]. Thus, future studies should further evaluate the impact of this technology in clinical practice.

### Antithrombotic strategies

Generally, anticoagulation in the first months after bioprosthesis implantation shall mitigate the risk of thromboembolic events until the valve prosthesis is fully endothelialized [30]. While current ESC and AHA/ACC guidelines recommend the use of oral anticoagulation with a vitamin K antagonist (VKA) for 3 months and 3 to 6 months, respectively, after surgical bioprosthetic MV replacement [30, 31], there is a lack of recommendations for antithrombotic treatment strategies following TMVR. Importantly, an elevated thrombosis rate (6% to 8%) was noted at different time points after Tendyne (under antiplatelet therapy) [32•], HighLife (under subtherapeutic anticoagulation) [33], and Fortis (Edwards Lifesciences, Irvine, CA, USA, under VKA plus antiplatelet therapy) device implantation [34], thus the latter one has been withdrawn from clinical use due to the issues with device thrombosis [33]. Indeed, the relatively large size of TMVR prothesis may increase thrombogenicity compared with surgical MV replacement or TAVR. In addition to the design, material and anchoring of TMVR protheses, the interaction between the native and transcatheter valves, patient related factors such as the presence of atrial fibrillation, hypercoagulable status, and left ventricle dysfunction determine thrombogenicity [33]. Considering to date clinical experience, anticoagulation with VKA in the first three months after TMVR procedure seems reasonable in patients who do not have an indication for long-term anticoagulation to diminish the risk of device thrombosis [32•, 33, 35]. An additional antiplatelet therapy could be applied in consideration of the patient’s individual bleeding risk as an empirical strategy [33]. Stringent clinical and TTE imaging follow-up is recommended to detect the occurrence of thrombosis. Follow-up screening with TEE might be beneficial in patients at higher risk of detecting subclinical TMVR thrombosis [36, 37]. However, future studies are needed to distinguish the ideal antithrombotic approach following TMVR, also further underscoring the appropriability of direct oral anticoagulants, as the RIVER trial has recently yielded the non-inferiority of rivaroxaban compared to VKA in patients with atrial fibrillation and a bioprosthetic MV [38].

## Devices and clinical experiences

There are numerous devices which have been developed for TMVR. However, most of these are at the early phase of development and have not yet received Food and Drug Administration (FDA) approval or the CE (Conformité Européenne) Mark. Thus, they are applied in studies or for compassionate use. The Tendyne valve is the first one that has received a CE mark approval. Others are expected to follow, whereas clinical use of some TMVR systems has been stopped due to economic or safety reasons, for example, Caisson (LivaNova, London, UK) and Fortis (Edwards Lifesciences, Irvine, CA, USA). Devices in clinical use are summarized in Table 1.

**Table 1:** Transcatheter mitral valve replacement devices with clinical experiences

Valve name	Manufacturer	Frame	Approach	Anchoring	Size
CardiAQ [39]	Edwards Lifesciences	Self-expanding, nitinol	Transapical, transseptal (31 Fr)	circumferential anchors on the ventricular and atrial side	30 mm
Intrepid [40]	Medtronic	Dual self-expanding nitinol stent	Transapical (35 Fr)	“champagne cork-like” effect, frictional elements on outer stent engage leaflets	27 mm, (fixation frame, available in three sizes: 43, 46, and 50 mm)
HighLife [41]	HighLife Medical	Self-expanding, nitinol	Transapical (39 Fr), (transseptal implantation of the ring [18 Fr])	“valve in ring” technique	31 mm
Tiara [42]	Neovasc	D-shaped self-expanding, nitinol	Transapical (32 Fr or 36 Fr)	3 ventricular anchors (2 anterior and 1 posterior)	35 mm or 40 mm
Tendyne [43]	Abbott Laboratories	Double frame, self-expanding, nitinol	Transapical (34 Fr)	Apical tether	30 – 43 mm anteroposterior x 34 - 50mm commissure-to-commissure
AltaValve [44, 45]	4C Medical Technologies	Spherical self-expanding, nitinol	Transapical (32 F)	supra-annular position, overexpansion fit, PET-skirt at the bottom enhances endothelization and tissue ingrowth	27 mm
EVOQUE [46]	Edwards Lifesciences	Self-expanding, nitinol	Transseptal (28 Fr)	circumferential anchors on the ventricular and atrial side	44 mm and 48 mm
SAPIEN M3 [47]	Edwards Lifesciences	Balloon-expanding, nitinol	Transseptal (20 Fr)	Nitinol docking system	29 mm
Cardiovalve [48]	Cardiovalve Ltd.	Self-expanding, nitinol	Transseptal (28 Fr)	24 anchors at ventricular side	40 to 50 mm

### Transapical approaches

#### CardiAQ

The CardiAQ prothesis (Edwards Lifesciences, Irvine, CA, USA) is a non-recapturable, self-expanding, nitinol trileaflet bovine pericardial valve, which was the first dedicated device for TMVR implanted transeptally (catheter size 31 Fr) in 2012 [39]. The device can be implanted transapically as well, and has two sets of circumferential anchors on the ventricular and atrial side to retain the valve in the mitral annulus. The ventricular anchors are positioned behind the MV leaflets and subvalvular apparatus, maintaining the chords and using native leaflets as support. To minimize LVOTO, the symmetrical valve is supra-annularly positioned. Moreover, the frame is covered by a polyester fabric skirt to reduce the risk for paravalvular leak. First results of the RELIEF (Reduction or Elimination of Mitral Regurgitation in Degenerative or Functional Mitral Regurgitation With the CardiAQ-Edwards Transcatheter Mitral Valve) study (NCT02722551), with 13 patients treated under compassionate use, yielded a technical success of 12/13 (92.3%) but high all-cause 30-day mortality of 7/13 (53.8%) [49]. Edwards Lifesciences has withdrawn the trial and presented a new redesigned device that was renamed the EVOQUE mitral valve replacement system and is described below.

#### Intrepid

**Fig. 1:**
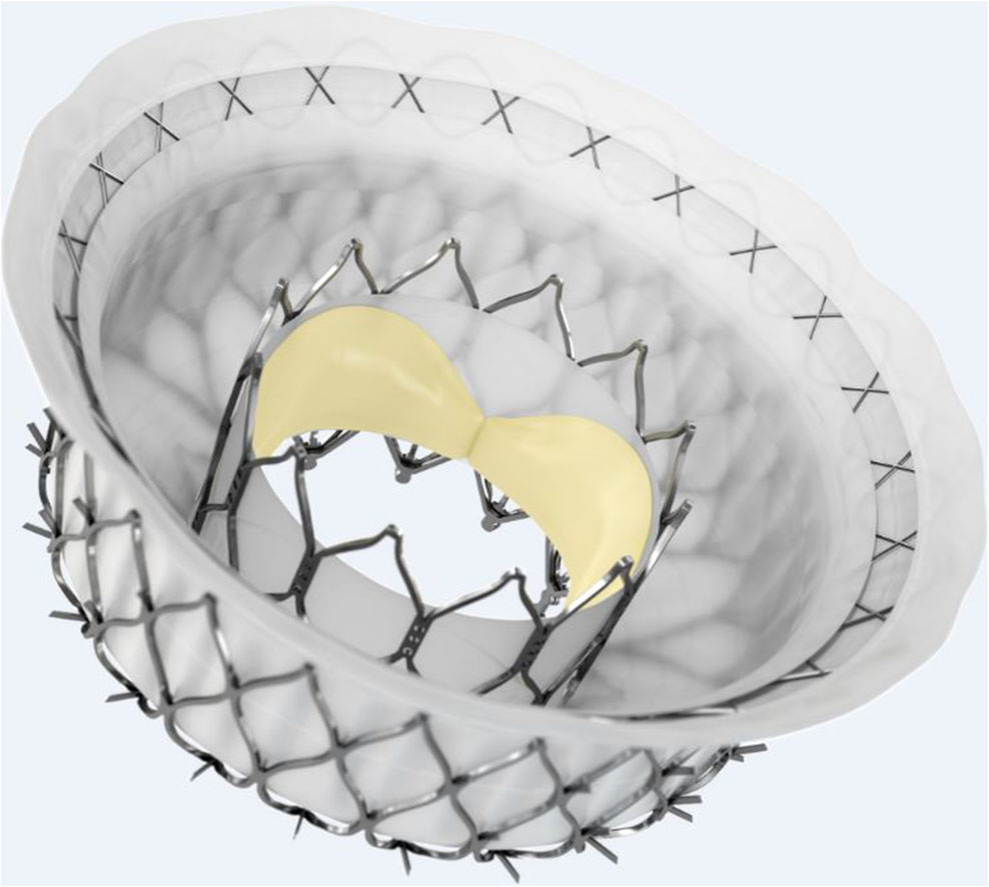
Intrepid prosthesis (Medtronic Inc.)

The Intrepid (Fig. 1) TMVR system (Medtronic, Minneapolis, MN, USA) consists of a symmetrical dual nitinol self-expanding stent, containing an outer stent frame (fixation frame, available in three sizes: 43, 46, and 50 mm) that has a flexible atrial portion, allowing conformability with the native mitral annulus [40]. The stiffer ventricular part is wider than the native annulus, enabling the mentioned “champagne cork-like” effect generated by a radial force along the valve stent to diminish the risk of LVOTO. The inner stent frame includes a 27 mm trileaflet bovine pericardium valve [40, 50]. Circumferential positioned frictional elements on the outer stent further fixate the prosthesis. The native leaflets and chordae are to be preserved and leveraged to seal around the device [51]. To date, the device is implanted via transapical access (35 Fr) under rapid ventricular pacing, and the system has been improved to be recapturable up to the point of final release [18]. The early experience has been published by Bapat et al. in 2018 [52]. Enrolling 50 patients with severe MR and at a very high risk for surgery, the technical success rate was 98%; 30-day mortality rate was 14%, with no disabling strokes [52]. During the follow-up (median 173 days), 11 patients (22%) died due to a cardiovascular cause [52]. With respect to complications, bleeding was the most common and mostly related to the access site [52]. Furthermore, echocardiography displayed mild or no residual MR in all patients who received the device [52]. The ongoing APOLLO trial (NCT03242642) will evaluate the safety and efficacy of Intrepid TMVR compared to conventional mitral valve surgery in patients with severe MR at high operative risk. Importantly, the FDA approved an early feasibility study for the new transfemoral applicable Intrepid at the end of 2019, which, considering the mentioned bleeding complications, could improve the safety profile of the Intrepid TMVR system.

#### HighLife

**Fig. 2:**
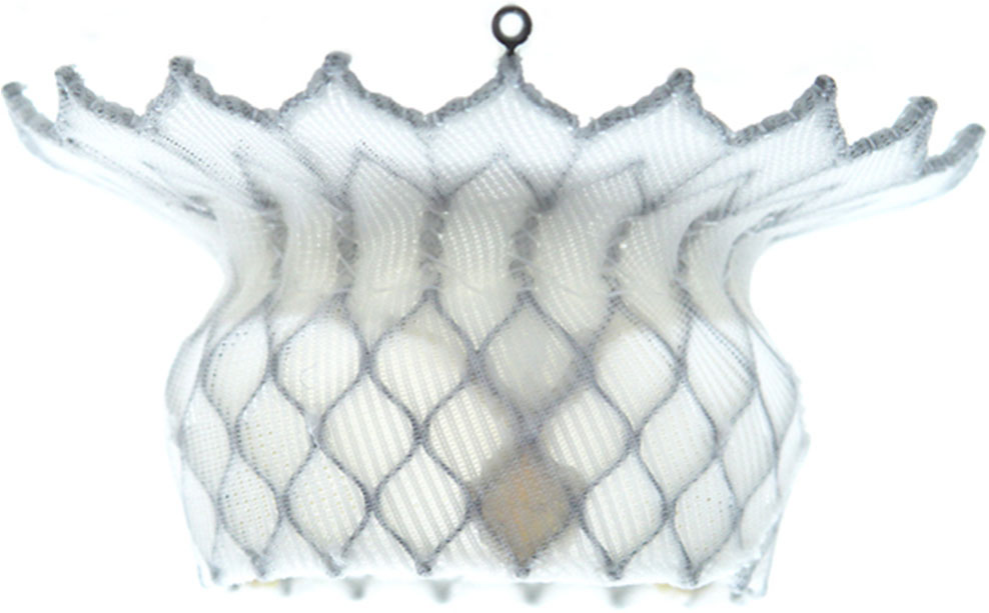
Highlife prosthesis (Highlife)

The HighLife (Fig. 2) TMVR system (HighLife Medical, Paris, France) is based on a “valve in ring” technique whereby the fixating ring is implanted via the transfemoral approach (18 Fr) in a subannulary position, while the symmetrical polyester covered nitinol trileaflet bovine 31 mm valve prosthesis is placed inside the ring by transapical access (39 Fr) [41]. Therefore, the native leaflets are caught between the ring implant and the prosthetic valve [53], fixing the anterior mitral leaflet and preserving LVOTO [54]. A systematic review by Del Val et al. yielded a technical success rate of 72.7% and procedure-related mortality of 18.2% in a cohort of 15 patients [55••]. One fatal case of LVOTO occurred, while stroke was not reported [55••]. A feasibility study of a novel transeptally delivered HighLife 28 mm prosthesis in patients with severe MR at high surgical risk is currently recruiting (NCT04029363).

#### Tiara

**Fig. 3:**
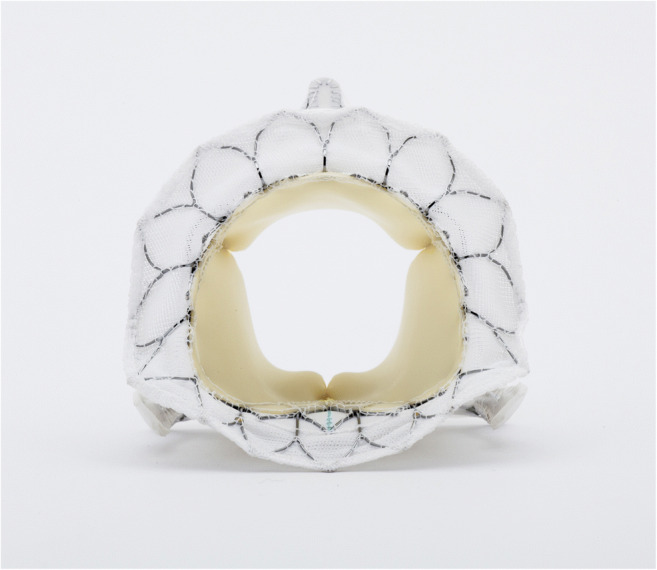
Tiara prosthesis (Neovasc)

The Tiara TMVR (Fig. 3) (Neovasc, Richmond, BC, Canada) is a trileaflet bovine pericardial prosthesis with a self-expanding nitinol frame. The frame is D-shaped, with a ventricular part equipped with three anchors (two anterior and one posterior) and an atrial part with an asymmetric skirt, sealing the prosthesis into the atrial segment of the mitral annulus [42]. The tapered-shaped prosthesis is available in two sizes: 35 mm and 40 mm. Tiara TMVR is delivered transapically (32 Fr or 36 Fr, respectively), whereby careful 3D TEE assessment is crucial to navigate the valve and ensure an optimal alignment of the D-shaped prosthesis in the MV annulus. Repositioning and retrieval can be securely performed prior to the release of the ventricular skirt. The early feasibility trial TIARA I and additional published cases of ongoing TIARA II trial (in total n=73) displayed a procedural success rate of 93% and a 30-day mortality of 11.2%, while 88% of the patients were free of relevant mitral regurgitation after the procedure [56]. LVOTO has not been reported, while stroke occurred in 5.4%, and access site complication in 6.9% of the cases [55••]. Thus, development of a transseptal deliverable Tiara TMVR system would be a substantial benefit to this system.

#### Tendyne

**Fig. 4:**
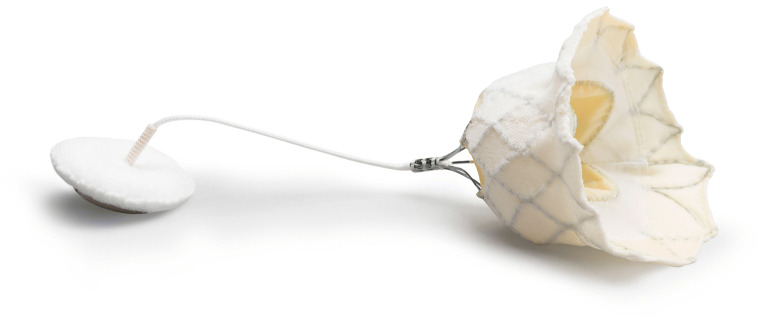
Tendyne prosthesis (Abbott Vascular)

The Tendyne (Fig. 4) TMVR system (Abbott Laboratories, Abbott Park, IL, USA) consists of a transapically delivered (34 Fr) trileaflet porcine pericardial valve on a self-expanding nitinol double frame stent and adjustable tether with an apical fixation pad [43]. While the outer stent is available in multiple sizes and D-shaped to adapt to the anatomy of the mitral annulus, the inner stent has a circular shape [18]. The valve is attached to the LV apex by a the epicardially fixed locking pad. An atrial cuff aids to further anchor the valve, avoiding prosthesis entering the ventricle when the tether is tensioned [11]. Remarkably, the Tendyne system is at any time fully retrievable, without need for rapid pacing during deployment [57]. While the first-in-man implantation of the first-generation was done in 2013 [58], the second-generation of the Tendyne TMVR system, which features a lower-profile valve to minimize LVOTO, was introduced in 2017 [59]. The initial feasibility study including 100 patients yielded a technical success rate of 96%, 30-day all-cause mortality of 6%, and 1-year all-cause mortality of 26% [32•]. One instance of major apical bleeding and three cases of stroke were reported [32•]. Furthermore, the Kansas City Cardiomyopathy Questionnaire score was improved by ≥10 points in 73% of the survivors [32•]. Accordingly, recent computed tomographic angiography analysis displayed a favorable LV remodeling in most patients treated with Tendyne TMVR, as closer proximity of the apical pad to the true apex predicted a favorable remodeling [60]. Thus, the clinical trial to evaluate the safety and efficacy of using the Tendyne MV system for the treatment of symptomatic mitral regurgitation (SUMMIT, NCT03433274) shall compare Tendyne TMVR with the MitraClip system. Because Tendyne TMVR has shown to be feasible in cases of severe mitral annular calcification (MAC) resulting in MR reduction with symptom improvement [61], a MAC cohort has been included in the SUMMIT trial as well.

#### AltaValve

The AltaValve TMVR system (4C Medical Technologies, Maple Grove, MN, USA) is composed of a self-expanding spherical-shaped nitinol stent with a 27 mm trileaflet bovine valve implanted in a supra-annular position, which may particularly diminish probability of LVOTO. A polyethylene terephthalate (PET)-skirt at the bottom of the frame should enhance endothelization and tissue ingrowth to prevent paravalvular leakage. To date, the system is implanted via a transapical approach (32-F), while a transseptal system is currently under development. First-in-human experiences showed the feasibility and procedural success of AltaValve TMVR with positive early clinical and valve hemodynamic outcomes [44, 45]. Thus, the AltaValve early feasibility study (NCT03997305) will further illuminate the safety and performance of the AltaValve for the treatment of moderate to severe or severe MR in patients who are considered high risk for mortality and morbidity from conventional open-heart surgery.

### Transseptal approaches

#### EVOQUE

**Fig. 5:**
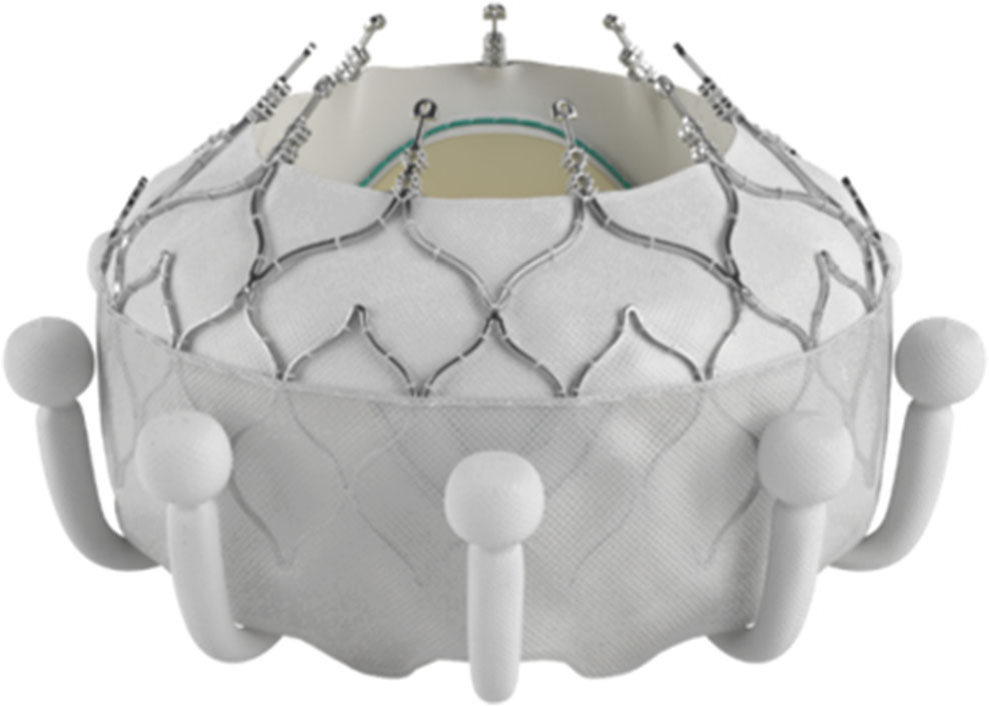
Evoque prosthesis (Edwards Lifesciences)

The EVOQUE (Fig. 5) (Edwards Lifesciences, Irvine, CA, USA) valve is available in two sizes (44 mm and 48 mm) that are compatible with a single delivery system. The low profile 28 Fr transseptal steerable delivery system enhances the maneuverability and depth control during a transfemoral–transseptal approach. A lower ventricular projection further minimizes LVOTO. A first in human experience in 14 patients treated with the EVOQUE mitral valve replacement system showed a technical success rate of 92.9% and a post-implantation MR reduction to mild or less in 92.9% of the patients. At 30 days, an all-cause mortality rate of 7.1% (one non-cardiovascular mortality case due to pneumonia) and two strokes (14.3%) were reported [46]. Including two patients with paravalvular leak closure, MR was mild or less in all implanted patients at 30 days, with no MR in 10 (83.3%) [46]. Further clinical studies are required to validate these first encouraging results, and the Edwards EVOQUE TMVR Early Feasibility Study for the treatment of clinically significant symptomatic MR (NCT02718001) is currently recruiting. The EVOQUE valve replacement system is now in trials for use in the tricuspid position.

#### Sapien M3

The SAPIEN M3 system (Edwards Lifesciences, Irvine, CA, USA) is based on the SAPIEN 3 TAVI system and consists of a PET-covered nitinol stent with a trileaflet bovine pericardial valve. Furthermore, the SAPIEN M3 TMVR system has a nitinol dock that encircles the chordae tendineae, securing the native MV leaflets and holds the valve in place [47]. The first-in-human study in patients with severe symptomatic MR at high surgical risk (n=10) showed technical success in 90%, total MR reduction to ≤ mild in 88.8% of implanted patients, no cases of death, and no stroke or LVOTO at 30 days [47]. Hence, the ENCIRCLE trial is currently recruiting to further establish the safety and effectiveness of the SAPIEN M3 system in patients with severe MR for whom surgical or TMVr options are deemed unsuitable (NCT04153292).

#### Cardiovalve

**Fig. 6:**
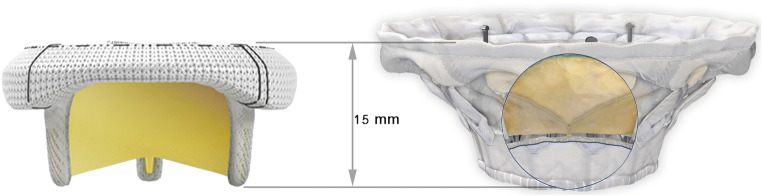
Cardiovalve prosthesis (Cardiovalve)

The Cardiovalve (Fig. 6) TMVR system (Cardiovalve Ltd., Or Yehuda, Israel) is composed of a self-expandable symmetrical trileaflet valve that includes both an atrial frame and a ventricular frame, delivered through a transseptal approach (28 Fr) [48]. The prosthesis is fixed into the mitral annulus by 24 anchors at the ventricular side [11]. It has a height of just 32 mm resulting in a low ventricular profile with no atrial protruding, thereby minimizing the risk for LVOTO [11]. Cardiovalve is available in three different size variations ranging from 40 to 50 mm [11]. First results of the ongoing AHEAD (European Feasibility Study of the Cardiovalve Transfemoral Mitral Valve System, NCT03339115) have shown a technical success rate of 100% (n=5) with reduction of MR but a high 30-day mortality rate of 60%. A 2-year follow-up of the first human case with the Cardiovalve TMVR system has recently been published, demonstrating good prosthesis function (mean gradient 4 mm Hg; no paravalvular leak) [48]. Therefore, further study results should be followed closely to evaluate the feasibility and risk profile of Cardiovalve TMVR.

## Conclusion

Untreated symptomatic MR is associated with a poor prognosis. Owing to demographic transition and subsequent increase of (frail) elderly patients, TMVR has emerged as a potential alternative to surgery. Numerous devices are under clinical assessment, and the early experience has shown high procedural success of TMVR. Because the technique faces several possible obstacles such as LVOTO after prosthesis deployment and access site complications, a careful preprocedural evaluation and dedicated patient selection is required. Future studies should address long-term prosthesis stability and degeneration, development of paravalvular leaks, and the risk of device thrombosis to distinguish optimal anticoagulation for each of the TMVR systems. Special attention will be paid to the evolution of TMVR prostheses suitable for less invasive transseptal implantation, as the transapical approach has been associated with poorer outcomes due to access site complications. Overall, TMVR could become a real alternative for patients with severe MR at high surgical risk.
